# Correction: Saberianpour et al. A Novel In Vitro Vascularized Dermis Organotypic Model of Acute and Chronic-like Wounds. *Cells* 2026, *15*, 485

**DOI:** 10.3390/cells15181653

**Published:** 2026-09-14

**Authors:** Shirin Saberianpour, Nadia Terrazzini, Matteo Santin

**Affiliations:** 1 Centre for Regenerative Medicine and Devices, University of Brighton, Huxley Building Lewes Road, Brighton BN2 4GJ, UK; s.saberianpour@brighton.ac.uk (S.S.); n.terrazzini@brighton.ac.uk (N.T.); 2 School of Applied Sciences, University of Brighton, Huxley Building Lewes Road, Brighton BN2 4GJ, UK

**References**

There were some errors in the original publication [1]. Some references were incorrectly included in our endnote database, resulting in incorrect citations of some references. We have replaced them with the following references. With these corrections, the order of some references has been adjusted accordingly:

4. Zhao, R.; Liang, H.; Clarke, E.; Jackson, C.; Xue, M. Inflammation in Chronic Wounds. *Int. J. Mol. Sci.* **2016**, *17*, 2085. https://doi.org/10.3390/ijms17122085.

25. Ma, Y.; Liu, Z.; Miao, L.; Jiang, X.; Ruan, H.; Xuan, R.; Xu, S. Mechanisms underlying pathological scarring by fibroblasts during wound healing. *Int. Wound J.* **2023**, *20*, 2190–2206. https://doi.org/10.1111/iwj.14097.

26. Costantini, E.; Aielli, L.; Serra, F.; De Dominicis, L.; Falasca, K.; Di Giovanni, P.; Reale, M. Evaluation of Cell Migration and Cytokines Expression Changes under the Radiofrequency Electromagnetic Field on Wound Healing In Vitro Model. *Int. J. Mol. Sci.* **2022**, *23*, 2205. https://doi.org/10.3390/ijms23042205.

27. Hosty, L.; Heatherington, T.; Quondamatteo, F.; Browne, S. Extracellular matrix-inspired biomaterials for wound healing. *Mol. Biol. Rep.* **2024**, *51*, 830. https://doi.org/10.1007/s11033-024-09750-9.

28. Mansour, M.R.; Ashour, N.A.; Gaballah, A.M.; Elzoghby, Y.E.; Elbatawy, R.M.; Shoraba, M.; Shoulah, S. Comprehensive Histopathological and Immunohistochemical Insights into Wound Healing from Cellular Dynamics to Translational Applications. *Adv. Anal. Pathol.* **2025**, *1*, 64–83. https://doi.org/10.17582/journal.aap/2025/1.64.83.

29. Xue, M.; Jackson, C.J. Extracellular Matrix Reorganization During Wound Healing and Its Impact on Abnormal Scarring. *Adv. Wound Care* **2015**, *4*, 119–136. https://doi.org/10.1089/wound.2013.0485.

30. Valero, C.; Javierre, E.; García-Aznar, J.M.; Menzel, A.; Gómez-Benito, M.J. Challenges in the Modeling of Wound Healing Mechanisms in Soft Biological Tissues. *Ann. Biomed. Eng.* **2014**, *43*, 1654–1665. https://doi.org/10.1007/s10439-014-1200-8.

31. Maver, T.; Gorenjak, M.; Marković, L.; Maver, U.; Kleinschek, K.S.; Glogovšek, M.; Smid, W. Combining 3D printing and electrospinning for preparation of pain-relieving wound-dressing materials. *J. Sol-Gel Sci. Technol.* **2018**, *88*, 33–48.

32. Patel, A.; Jain, P.; Ajazuddin. Recent advances in the therapeutics and modes of action of a range of agents used to treat ulcerative colitis and related inflammatory conditions. *Inflammopharmacology* **2025**, *33*, 4965–4996. https://doi.org/10.1007/s10787-025-01906-8.

33. Freedman, G.; Entero, H.; Brem, H. Practical treatment of pain in patients with chronic wounds: Pathogenesis-guided management. *Am. J. Surg.* **2004**, *188*, 31–35. https://doi.org/10.1016/s0002-9610(03)00289-7.

34. Kunz-Schughart, L.A.; Schroeder, J.A.; Wondrak, M.; van Rey, F.; Lehle, K.; Hofstaedter, F.; Wheatley, D.N. Potential of fibroblasts to regulate the formation of three-dimensional vessel-like structures from endothelial cells in vitro. *Am. J. Physiol. Physiol.* **2006**, *290*, C1385–C1398. https://doi.org/10.1152/ajpcell.00248.2005.

35. Cutter, S.; Wright, M.D.; Reynolds, N.P.; Binger, K.J. Towards using 3D cellular cultures to model the activation and diverse functions of macrophages. *Biochem. Soc. Trans.* **2023**, *51*, 387–401. https://doi.org/10.1042/bst20221008.

36. Tremel, A.; Cai, A.; Tirtaatmadja, N.; Hughes, B.; Stevens, G.; Landman, K.; O’cOnnor, A. Cell migration and proliferation during monolayer formation and wound healing. *Chem. Eng. Sci.* **2009**, *64*, 247–253. https://doi.org/10.1016/j.ces.2008.10.008.

37. Jabbari, P.; Kim, J.H.; Le, B.H.; Zhang, W.; Zhang, H.; Martins-Green, M. Chronic Wound Initiation: Single-Cell RNAseq of Cutaneous Wound Tissue and Contributions of Oxidative Stress to Initiation of Chronicity. *Antioxidants* **2025**, *14*, 214. https://doi.org/10.3390/antiox14020214.

38. Bini, F.; D’aLessandro, S.; Agarwal, T.; Marciano, D.; Duchi, S.; Lucarelli, E.; Ruocco, G.; Marinozzi, F.; Cidonio, G. Biomimetic 3D bioprinting approaches to engineer the tumor microenvironment. *Int. J. Bioprinting* **2023**, *9*, 1022. https://doi.org/10.36922/ijb.1022.

**Text Correction**

Due to some adjustments made to the references, corrections have been made to the following paragraphs. In addition, references previously numbered as n. 39 and 40 have been deleted.

**Discussion**

The present study successfully established, for the first time, an organotypic co-culture in vitro model that is capable of simulating the early phases of an acute and chronic wound state by using the biomimetic substrate PhenoDrive-Y. The model recapitulated many of the pathological hallmarks of chronic wounds, including impaired fibroblast migration, compromised angiogenesis, disrupted deposition of the ECM components, and inflammatory cell phenotypic changes. The alteration of these processes was shown to be partially reversed following therapeutic intervention by the application of clinically available wound dressings. Together, these findings address an important gap in modelling wounds by providing a stable, reproducible in vitro platform of enhanced translational relevance.

These results confirmed that PhenoDrive-Y supported the formation of organized tissue-like structures on a 2D plane without the need for a gel encapsulation process, whereas traditional tissue-culture plastic (TCP) lacks both structural and biochemical cues necessary to promote organotypic cultures. Especially important is the capability of PhenoDrive-Y to maintain architectural stability for >72 h. This was a significant advantage considering that many natural hydrogels, including Matrigel, degrade rapidly, making it difficult to employ them in studies exceeding three days [23,24]. The ability of PhenoDrive-Y to induce tissue-like structures has been ascribed to the ordered presentation of integrin bioligands, as well as to its demonstrated mesh-like topography [24]. The incubation time after scratching was deliberately limited to 24 h in the early phase of the study to enable the observation of the early phases of reorganization of the tissue-like structures while enhancing the opportunity to identify any difference occurring between the acute and chronic wound-like conditions. In the case of the model validation by testing of wound dressings, the period of observation after scratch was extended to a further 48 h to maximize any effect produced by the dressings and to simulate as closely as possible the time of application of dressings that in clinics varies from 2 to 3 days, unless diabetic ulcers are treated.

In comparison with the acute wound-like model and the control, the chronic wound model showed markedly reduced fibroblast migration and closure of the gap generated by a conventional mechanical scratch. This is consistent with clinical pathology, where fibroblasts in chronic ulcers exhibit stalled motility and persist in a “non-healing” state [25]. This decreased migration corresponds with that seen by Costantini et al. to show reduced migration capability under conditions of sustained pro-inflammatory cytokines [26]. These sustained pro-inflammatory conditions were simulated in this study by deliberately spiking the cells with cytokines concentration above the levels observed during the clinical studies of chronic ulcers (high rather than low nanogram scale). The observed angiogenic defects were characterized by reduced tube length and fewer branching points; this further reflects the in vivo chronic wound environment, where cytokine-induced cytotoxicity leads to endothelial dysfunction [15].

The observation of thin, fragmented collagen fibers and dysregulated hyaluronic acid distribution in ECM deposition patterns within the chronic wound model is supported by their significantly higher levels in the supernatants, reflecting the pathological features observed in non-healing human ulcers [27,28]. The initial organization of collagen bundles in the acute wound onset model is consistent with the successful transition of the cells into a proliferative phenotype and the onset of the wound-contracting phase, as observed in vivo during normal acute wounds healing [29,30]. Furthermore, the impaired collagen architecture within the chronic model was similar to the findings of Maver et al. who demonstrated ECM disorganization in hydrogels that were exposed to IL-6 and TNF-α to mimic chronic wound conditions [31]. Likewise, under conditions simulating a high inflammatory status, the cells appeared to synthesize GAGs, but these components were not released in the extracellular matrix and remained mainly localized within the cells or in their immediate surroundings.

Interestingly, macrophage polarization results showed no significant difference between the acute and chronic wound models 24 h after scratch induction and monocyte seeding, despite cytokine induction. This is perhaps reflective of the short induction period and would suggest that longer exposure may indeed be required for macrophage phenotypic divergence. This finding is supported by studies from Lacina et al. which suggested that exposure longer than 48 h was required to drive a more stable M1 dominance under chronic inflammatory conditioning [18,19]. However, following therapeutic treatment by wound dressings, iNOS expression was reduced, and CD206 increased, with the strongest response observed in the acute wound model. This confirms that the system is responsive and capable of demonstrating phenotype reversal in inflammatory cells even without prior stimulation with phorbol merystate acetate (PMA) followed by IL-4 incubation. Indeed, the spiking of U937 with PMA and IL-4, which is typically employed to differentiate monocytes first into macrophages and then into the CD206-expressing M2 phenotype, was deliberately omitted in this study to ensure that any observed differentiation was driven by the microenvironment and by the contact with the wound dressings.

Treatment with N-A showed clear differences in healing potential when tested under the two conditions. The acute wound model demonstrated almost complete closure of the gap by fibroblasts within 48 h of scratch induction, along with enhanced deposition of ECM components, and evidence of macrophage repolarization. In contrast, when applied to the chronic wound model, this dressing presented only minimal improvements in the studied healing parameters, highlighting the clinical challenge of reverting long-standing chronic wound inflammation. This aligns with observations by Patel et al. and Freedman et al., who reported reduced therapeutic responsiveness in chronic ulcers [32,33]. The stimulation of all the tested parameters (i.e., angiogenesis, fibroblast organization around angiogenic sprouting and deposition of ECM components) was more evident in the case of the alginate-based dressing, Kaltostat, suggesting a scaffolding or metabolic effect of this biodegradable polysaccharide-based (i.e., alginate) dressing in tissue cells. At the same time, given the limitation of the cell counts, particularly in the case of cluster formation, the changes observed may be more cautiously interpreted as caused by differences in iNOS-labeling intensity and responsiveness to the dressings’ foreign body character rather than a fully resolved M1/M2 shift.

Few existing models have integrated fibroblasts, endothelial cells, and macrophages into one 3D structure without cell encapsulation into a gel or scaffold [34,35]. Most past models of chronic wounds depend on either the basic cell monolayer scratch assays [36] or hydrogels that degrade fast [24] or systems of a single cell type [37]. While aligning with the more advanced multicellular systems such as those described by Macedo et al. and Maver et al. [20,31,38], the wound models reported here offer improved reproducibility and the potential of dissecting specific biochemical and cellular pathways in acute and chronic wound states in their early phase. For the purpose of model standardization across laboratories, cell lines of both fibroblasts and monocytes were preferred to primary cells that are known to be prone to donors’ variabilities. While it is acknowledged that the lung fibroblasts and HUVECs used in this study do not tightly recapitulate the dermis cell populations, their widespread use and relative ease of culturing have been preferred to maximize the wide adoption of the model. Indeed, in addition to their suitability for studies of cellular and biochemical pathways of wound healing, these in vitro organotypic models also represent a meaningful step towards the standardized and reproducible preclinical screening of novel therapeutics and medical devices.

The authors state that the scientific conclusions are unaffected. This correction was approved by the Academic Editor. The original publication has also been updated.
